# Effectiveness of Transitional Care Program among High-Risk Discharged Patients: A Quasi-Experimental Study on Saving Costs, Post-Discharge Readmissions and Emergency Department Visits

**DOI:** 10.3390/ijerph20237136

**Published:** 2023-12-02

**Authors:** Moonseong Heo, Kevin Taaffe, Ankita Ghadshi, Leigh D. Teague, Jeffrey Watts, Snehal S. Lopes, Peter Tilkemeier, Alain H. Litwin

**Affiliations:** 1 Department of Public Health Sciences, Clemson University, Clemson, SC 29634, USA; 2 Department of Industrial Engineering, Clemson University, Clemson, SC 29634, USA; 3 Department of Medicine, Prisma Health, Greenville, SC 29605, USA; 4 Value-Based Care & Network Services, Prisma Health, Greenville, SC 29605, USA; 5 Department of Medicine, University of South Carolina School of Medicine—Greenville, Greenville, SC 29605, USA; 6 School of Health Research, Clemson University, Greenville, SC 29634, USA

**Keywords:** post-discharge care, transitional care program, cost, hospital readmission, ED visits

## Abstract

Transitional care programs (TCPs), where hospital care team members repeatedly follow up with discharged patients, aim to reduce post-discharge hospital or emergency department (ED) utilization and healthcare costs. We examined the effectiveness of TCPs at reducing healthcare costs, hospital readmissions, and ED visits. Centers for Medicare and Medicaid Services Bundled Payments for Care Improvement (BPCI) program adjudicated claims files and electronic health records from Greenville Memorial Hospital, Greenville, SC, were accessed. Data on post-discharge 30- and 90-day ED visits and readmissions, total costs, and episodes with costs over BPCI target prices were extracted from November 2017 to July 2020 and compared between the “TCP-Graduates” (N = 85) and “Did Not Graduate” (DNG) (N = 1310) groups. As compared to the DNG group, the TCP-Graduates group had significantly fewer 30-day (7.1% vs. 14.9%, *p* = 0.046) and 90-day (15.5% vs. 26.3%, *p* = 0.025) readmissions, episodes with total costs over target prices (25.9% vs. 36.6%, *p* = 0.031), and lower total cost/episode (USD 22,439 vs. USD 28,633, *p* = 0.018), but differences in 30-day (9.4% vs. 11.2%, *p* = 0.607) and 90-day (20.0% vs. 21.9%, *p* = 0.680) ED visits were not significant. TCP was associated with reduced post-discharge hospital readmissions, total care costs, and episodes exceeding target prices. Further studies with rigorous designs and individual-level data should test these findings.

## 1. Introduction

### 1.1. Backgrounds

Globally, hospitals face the issue of readmissions of patients with heart failure, acute myocardial infarction, or pneumonia. Patients are readmitted to the hospital if conditions like loss of strength, mobility, nutritional deficits, new impairments, or sleep deprivation are observed within 30 days of discharge. Such readmissions cause a burden on healthcare resources and increase the cost of readmissions [1]. Hospital readmission is defined as the unplanned admission of a patient to a hospital within a specific discharge period after an initial stay at the hospital [2]. For instance, readmission to the hospital within 30 days of discharge accounts for nearly 20% of hospitalized older Medicare patients [3]. Some adverse events (e.g., diagnostic and therapeutic errors, as well as common and potentially harmful adverse drug events), often leading to hospital readmissions, are expected to occur after discharge [4].

Many state and national policies include incentives for improving transitional care and disincentives for readmission. In 2015, the Center for Medicare and Medicaid Services (CMS) announced financial penalties of up to 1% of Medicare reimbursements to more than 2000 hospitals due to high readmission rates [5]. The penalty applies to the following six health conditions with a high 30-day readmission rate: acute myocardial infarction (AMI), heart failure (HF), pneumonia (PNE), chronic obstructive pulmonary disease (COPD), total hip/knee arthroplasty (HK), and coronary artery bypass graft (CABG) surgeries [2].

### 1.2. Research Gap in the Literature

As part of the transitional care programs (TCPs), which have been developed to reduce post-discharge acute hospital or emergency department (ED) utilization, hospital care team members repeatedly follow up with discharged patients so that patients with critical health conditions get the care they require [5,6,7]. TCPs provide customized post-discharge follow-up care to patients with the most critical chronic health conditions that typically require the highest care and incur the greatest cost [8,9]. The essential transitional care parameters for high-need and high-cost patients include targeting patients during transitions, home visits, rapid outpatient follow-up, and medication management [10,11]. Studies on diverse types of TCPs employed by various healthcare systems have provided evidence suggesting that TCPs may be beneficial for reducing healthcare utilization and costs [12,13,14]. However, besides heart and neurological conditions [5,12,14], little documented evidence exists to assess program success within subgroups of other common high-need and high-cost conditions. Also, few studies that we are aware of have assessed the effectiveness of TCPs on the outcome of keeping healthcare costs within the limit of target prices.

### 1.3. Study Aims

In an effort to allow us to improve patient outcomes following hospital discharge, a TCP was initiated for patients with a high risk of hospital readmissions and ED visits. The primary aim of this study was to test whether the TCP was more effective in reducing avoidable hospital readmissions and ED visits compared to other types of post-discharge visits or no follow-up. A secondary aim was to examine if TCP care reduced health care costs compared to other types of visits or no follow-up. All of the comparisons were made between different types of post-discharge care for each health condition that required hospitalization for inpatient treatments.

## 2. Materials and Methods

### 2.1. Study Design, Setting, and Population

This was a quasi-experimental observational quality improvement study based on a retrospective electronic health record (EHR) review, as opposed to a randomized trial, analyzing data collected from patients discharged between December 2017 and July 2020 from the Greenville Memorial Hospital in Greenville, SC, USA, which is the largest of all facilities that comprise the Prisma Health system.

### 2.2. Data Sources

Data were extracted not only from the Prisma Health electronic health record (EHR) database but also from claims files adjudicated by the Centers for Medicare and Medicaid Services (CMS) Bundled Payments for Care Improvement (BPCI) program. Claims data for this program allow us to capture a more complete and comprehensive picture of care that includes all of a beneficiary’s healthcare utilization and spending, including care provided outside of Prisma Health EHR, a Level 1 trauma center in the Southeast United States. Non-identifiable aggregate data (as opposed to individual-level data) were extracted. We note that the BPCI program requires the destruction of beneficiary-level data after the program’s conclusion, and thus, any individual-level data used in this study are not recoverable. The aggregate data were available for several diagnostic medical conditions [15], including COPD [16,17,18], sepsis [19,20], percutaneous coronary intervention (PCI) [21], chronic heart failure (CHF) [22,23,24,25], and pneumonia (PNE) [26,27]. As part of the BPCI program, we selected this set of conditions for which we assume full financial risk. The study protocol was approved by the institutional review board of Prisma Health.

### 2.3. Episodes for Index Hospitalization

The patients observed after discharge as part of the study were treated for one of the following five diagnoses for hospitalizations: CHF, COPD, PNE, sepsis, or PCI. In this study, the ‘index’ episode refers to the hospitalization that served as the basis for defining care cost, post-discharge care types, readmissions, and ED visits. Although a patient could have multiple hospitalization episodes, the episodes (as opposed to patients) served as the unit of analysis. In other words, the sample sizes for the aggregate statistics, such as average costs, were determined based on the number of index episodes, not the number of patients.

### 2.4. The Implemented Transitional Care Program (TCP)

The TCP being evaluated in this study was a structured transition clinic including multidisciplinary healthcare team members, and frequent and timely patient visits. The team included a pharmacist, a nurse, social workers, and the physician who saw the patient at each visit. The first visit was typically within seven days following discharge but had to occur within the first 14 days post-discharge. Providers overseeing patient discharge referred patients to the TCP. The decision about whether to refer to the TCP was based on the provider’s clinical judgment about the benefit the program could have for the patient. However, the intent was to offer the TCP to all BPCI participants. A team member called the patients to offer the TCP. Once the patient had elected to enroll, the patient was introduced to the program during discharge or within two business days after discharge and given the opportunity for enrollment. Patients were then seen once weekly for four weeks. The patient had to be seen four times to “graduate”. If a patient missed a visit, attempts were made to reschedule within the same business week. Although the majority of the four visits occurred within four weeks, all four visits occurred within six weeks. A few patients may be seen more than four times, depending on the patient’s needs. The TCP included the following characteristics: (1) a phone call within 48 h of discharge to provide real-time recommendations in collaboration with transitional care clinicians; (2) follow-up visits with the multidisciplinary team; (3) full medication reconciliation conducted by the pharmacist taking into account medications listed on the discharge summary, actual medications brought in by the patient, and drug–drug interactions; (4) provision of diabetes education if applicable; (5) social determinants of health (SDoH) screening and social work assessment to overcome SDoH; (6) team huddle involving nurses, pharmacists, and physicians prior to the physician seeing the patient; (7) clinicians providing the TCP were all hospitalists who are well trained and competent with managing high-acuity and medically complex patients; (8) focus on preventive vaccine care including influenza, COVID, pneumonia, and zoster vaccines to appropriate patients; (9) all in-person visits; (10) provision of transportation with taxis for those who were unable to arrange their own transportation; (11) availability of flexible scheduling; (12) individualized care with a minimum of 4 visits to qualify as “graduated” but allowing additional visits if medically indicated (and including an option for early discharge as well). The patients admitted to the hospital met the Bundled Payments for Care Improvement (BPCI) criteria to enroll in the TCP.

### 2.5. Types and Grouping of the Post-Discharge Care

The following post-discharge care groups were not randomly allocated but selected from existing data in the hospital Electronic Health Record (EHR) system for the present study. Again, the sample size for each group was determined based on the number of index episodes across all five diagnoses for hospitalizations.

TCP-Graduated group: This group was composed of patients who enrolled in the TCP and were discharged upon completion of the program (i.e., a minimum of four visits).

Did Not Graduate (DNG) group and its subgroups: All the other patients who did not graduate or did not enroll in the TCP were collectively referred to as the “Did Not Graduate” (DNG) group. Patients who declined to enroll in the TCP would be scheduled for an office visit with their Primary Care Provider (PCP), that is, either a specialized Transitional Care Management (TCM) visit or a standard Evaluation and Management (E&M) visit within 14 days of discharge. They would also need to have a documented phone call from the PCP nurse within 48 h of discharge. At a TCM visit, there had to be documentation of medical list reconciliation and documentation of the level of medical decision-making rated as low, moderate, or high risk. An E&M visit also occurred within 14 days of discharge. But in contrast to TCM, an E&M visit did not require a nursing phone call or the same protocolized visit as the TCM visit. The patients were recommended for the TCP-structured visit if they had risk factors for readmission based on clinical criteria. However, the patients could ultimately choose among any of the follow-up care models. The follow-up timelines were variable and based on patient preference and clinical criteria determined by the transition clinic care team.

**Figure 1:**
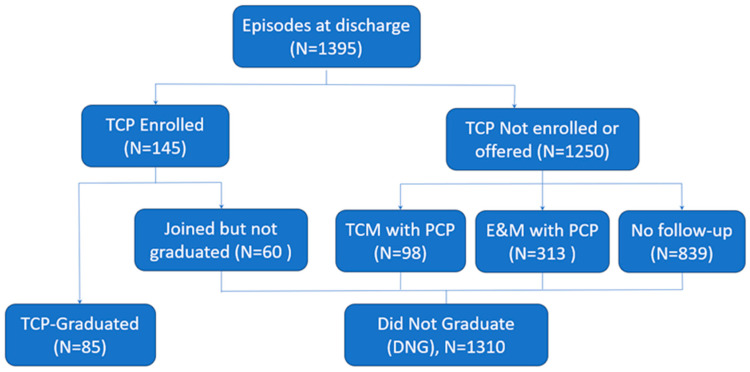
Types of post-discharge care groups. Abbreviations: TCP (Transition Care Program), TCM (Transitional Care Management), E&M (Evaluation and Management), PCP (Primary Care Provider).

Therefore, the collective DNG group can be broken down into four subgroups of patients. First, there were the patients who enrolled in TCP but did not complete the program, referred to as the “Joined but did not graduate” (Joined but DNG) group. Second, there were the patients in the “Transitional Care Management (TCM) visit” group who did not pursue the TCP but instead followed with a TCM visit with a primary care provider (PCP) within 14 days of discharge. This visit type allowed for a longer visit to review and coordinate care following discharge from the hospital by the PCP team. Third, there were the patients in the “Evaluation and Management (E&M) visit group” who did not pursue the TCP but instead followed with a standard E&M visit with PCP within 14 days of discharge. Finally, the “No follow-up” group comprised those patients who received no follow-up care (Figure 1).

### 2.6. Post-Discharge Utilization Outcomes

The post-discharge utilization outcomes compared between the post-discharge care groups were the frequency of the following four presentations at the hospital: 30-day readmission, 90-day readmission, 30-day ED visit, and 90-day ED visit. Although 30-day post-discharge outcomes are required for evaluations by the CMS, we also analyzed 90-day outcomes to evaluate the TCP as a part of an internal quality improvement project, which has also been undertaken in other studies [28,29,30,31]. Readmission is defined as being readmitted to the hospital for any condition, whereas an ED visit is defined as presenting at the emergency department for any condition without being readmitted. Even if some episodes had multiple utilizations for each of the outcomes of 30- and 90-day readmissions or ED visits for index episodes, the number of utilizations was counted as only one, i.e., dichotomized between ever- and never-utilized with scores 1 and 0, respectively, for each episode.

### 2.7. Cost Outcomes

The aggregate average total cost per hospitalization episode and its target prices were obtained by applying the BPCI advanced (BPCI-A) model methodology supported by the CMS. The total cost used in the BPCI-A model refers to the expenses that Medicare/the payer incurs for services, i.e., in the BPCI-A model, what is considered a cost to the payer is revenue for the provider in the form of reimbursements. Specifically, the total cost reflects the fee-for-service claims paid by Medicare rather than the actual cost incurred by hospitals/providers to provide the service. For example, all services provided within the 90-day episode that had a reimbursable Medicare claim are included in the total cost/reimbursement, such as payments for TCP visits. The total cost per episode for the present analysis was calculated as the sum of the initial index inpatient stay cost and 1–30- and 31–90-day post-discharge care costs. However, it did not include costs spent for items and services (e.g., transportation costs like taxi fares) identified as exclusions in the programs’ methodology. The average total costs per episode were computed for a combination of all conditions for hospitalizations as well as for each condition. The costs spent during 1–30 and 31–90 days following discharge were also available per episode. The total costs were calculated as the sum of costs over the multiple utilizations, if any, per index episode. The total costs/reimbursements were then reconciled against a target price. If the total cost/reimbursement exceeded this target price, providers were required to repay Medicare a portion of the difference. On the other hand, if the total cost/reimbursement was less than the target price, the provider received a bonus payment. The number of episodes whose total care cost exceeded the BPCI target prices was also obtained for each type of episode.

### 2.8. Statistical Analysis

Chi-square or Fisher’s exact tests, depending on the expected cell counts, were used to test the significance of differences in the post-discharge utilization outcomes across all initial hospitalization episode conditions between the post-discharge groups, as well as differences in percentages of episodes with costs over target prices. Since these overall analyses could not be conducted after adjusting for different conditions, we also conducted the same comparisons stratified by conditions. Specifically, seven between-group comparisons were made: Graduated vs. DNG; Graduated vs. Joined but DNG; Graduated vs. TCM visit; Graduated vs. E&M visit; Graduated vs. No follow-up; TCM visit vs. No follow-up; and E&M visit vs. No follow-up. Of note, statistical tests on the difference in amount of cost were not possible because only aggregate average costs per episode were collected for the reasons noted above. All statistical tests were performed using SAS v9.4 software, and all comparisons with a two-sided *p*-value less than 0.05 were considered statistically significant.

## 3. Results

### 3.1. Participant Compositions

Overall, a total of 1395 episodes were included in the study, of which 85 (6.1%) were episodes of patients who graduated from the TCP. Of the 1395 episodes, the “DNG” group included 1310 (93.9%) episodes in total, and among the “DNG” group, 839 (60.1%) episodes had no follow-up appointment. More details on the number of episodes stratified by post-discharge care groups and conditions for hospitalization are presented in Table 1.

### 3.2. Overall Comparisons of Utilization Outcomes

Table 2 presents the between-group comparisons of the post-discharge outcomes, combining all conditions for hospitalizations. The TCP-Graduated group had both significantly lower 30-day (7.1% vs. 14.9%, *p* = 0.046) and 90-day readmissions (15.5% vs. 26.3%, *p* = 0.025) compared to the collective DNG group. Compared to the no follow-up group, the TCP-Graduated group also had significantly lower 30-day (7.1% vs. 15.5%, *p* = 0.036) and 90-day (15.5% vs. 26.7%, *p* = 0.022) readmission rates. The TCP-Graduated group did not have significantly different 30-day (9.4 vs. 11.2%, *p* = 0.607) and 90-day (20.0% vs. 21.9%, *p* = 0.680) ED visits compared to the collective DNG group. When compared to DNG subgroups, the TCP-Graduated group did not have any significant differences in the rates of 30- or 90-day ED utilization outcomes.

### 3.3. Comparisons of Utilization Outcomes by Hospitalization Conditions

Table 3 presents the between-group comparisons for each condition for initial hospitalizations. The TCP-Graduated group had both significantly lower 30- (8.3% vs. 42.1%, *p* = 0.013) and 90-day (20.8% vs. 57.9%, *p* = 0.013) readmission rates compared to the “Joined but DNG” group for the CHF episodes. Notably, the TCP-Graduated group had no 30-day ED visits for both the COPD and sepsis episodes and no 30- or 90-day readmission or ED visits for the PNE episodes. The TCM visit group had significantly higher 90-day ED visits compared to the no follow-up group (44.8% vs. 21.3%, *p* < 0.001) for the PCI episodes.

### 3.4. Comparisons of Absolute Costs

The combined average total cost per episode was smaller for the TCP-Graduated group by USD 6194 compared to the collective DNG group (USD 22,439 vs. USD 28,633), saving 21.6% of the cost of the latter (Table 4). The 1–30-day post-discharge cost was much smaller by USD 4535 (or 48.9% saving) for the TCP-Graduated group (USD 4740 vs. USD 8708), and the 31–90-day post-discharge cost was also lower by USD 366 (or 5.6% savings) for the TCP-Graduated group (USD 6168 vs. USD 6534). Both the combined total cost and the 1–30-day cost of the TCP-Graduated group were lower compared to all of the DNG subgroups, although the 31–90-day cost was lower or higher depending on the types of care among the DNG subgroups (Table 4).

Except for the CHF condition, the total cost of the TCP-Graduated group was lower compared to the collective DNG group for all other conditions for hospitalization. Furthermore, both the 1–30-day and 31–90-day post-discharge costs were lower for the TCP-Graduated group for all conditions except for the CHF, for which the TCP-Graduated group had a much higher 31–90-day cost (USD 11,654 vs. USD 5848), yet a lower 1–30-day cost (USD 6054 vs. USD 7799) (Table 4). Further breakdowns of costs by the subgroups of the collective DNG group are also provided in Table 4.

### 3.5. Comparisons of Percentages of Episodes with Costs over Target Prices

The proportion of TCP graduates with costs over BPCI target prices was significantly lower compared to the collective DNG group (25.9% vs. 36.6%, *p* = 0.018) and the no follow-up subgroup (25.9% vs. 41.6%, *p* = 0.005) when all episodes were combined (Table 4). Similarly, within the sepsis condition, the proportion of TCP graduates with costs over BPCI target prices was significantly lower compared to the collective DNG group (13.6% vs. 36.0%, *p* = 0.031) and the No follow-up subgroup (13.6% vs. 41.0%, *p* = 0.010). Remarkably, the proportion of TCP graduates with costs over BPCI target prices was in general, smaller, albeit not significantly, than that of any other post-discharge care groups, except for a few comparisons across all episodes (Table 4).

## 4. Discussion

### 4.1. Summary of Findings

The primary finding from this study is that the TCP had a positive impact with respect to reducing post-discharge episode care cost and significantly reducing overall patients’ readmission rates for both 30 and 90 days following discharge compared to the collective DNG group. The overall 30- or 90-day ED visits per episode were also lower, yet not significantly, for the TCP-Graduated group vs. the collective DNG group. Furthermore, the effect of TCP on improving the post-discharge utilization outcomes appeared to be in general, independent of conditions for hospitalizations as supported by smaller, albeit not significant, percentages of all four post-discharge utilization outcomes across all conditions for hospitalizations. This finding is consistent with findings from other transitional care models in the literature, such as the Care Transitions Initiative (CTI) [28], Project Reengineering Discharge (RED) [29], Comprehensive Discharge Planning (CDP) [30], and the Transition Care Coordinator (TCC) model [31]. Each of these models has been successful in reducing hospital readmissions. Both CTI and CDP were designed specifically for elderly populations (65 and older, and 70 and older, respectively), and each involved in-home visits. Like the past models of RED and TCC, TCP did not restrict the patient population by age, and there were no in-home visits.

### 4.2. Review of Other Studies in the Literature

Under the CTI study [28], patients received (1) tools to promote cross-site communication, (2) encouragement to take a more active role in their care and to assert their preferences, and (3) continuity of care across settings and guidance from a “transition coach”. The study suggests that coaching chronically ill older patients and their caregivers to ensure that their needs are met during care transitions may reduce the rates of subsequent rehospitalization.

The RED study [29] utilized a nurse discharge advocate who worked with patients during their hospital stay to arrange follow-up appointments, confirm medication reconciliation, and conduct patient education with individualized instruction booklets sent to primary care providers. A clinical pharmacist called patients two to four days after discharge to reinforce the discharge plan and review medications. The intervention group had a significantly lower rate of hospital utilization.

The CDP study [30] examined the effects of a comprehensive discharge planning protocol designed for the elderly and implemented by nurse specialists on patient and caregiver outcomes and cost of care. Study findings support the need for CDP to improve their outcomes after hospital discharge and to achieve cost savings. Patients had fewer readmissions, fewer total days rehospitalized, lower readmission charges, and lower charges for health care services after discharge.

The TCC study [31] evaluated two forms of an evidence-based, multi-component transitional care intervention. A quasi-experimental evaluation design compared the outcomes of TCC Care to Usual Care. Nurse TCCs provided either a full intervention (delivered in-hospital and by post-discharge phone call) or a partial intervention (phone call only). TCC Care had significantly lower readmission rates and lower costs at both 30 and 90 days following discharge.

### 4.3. Outcome Differences among TCP-Graduated Group, DNG Group, and DNG Subgroups

Further investigation of outcome differences between the TCP-Graduated group and the DNG subgroups revealed that the differences between the TCP-Graduated group and the collective DNG group were driven by the joined but DNG and the no follow-up groups. Whether this difference was due to the effectiveness of the intervention or because the patients selecting themselves into these groups may have worse health needs further investigation. Also, given our results that the TCP-Graduated group and the TCM visit and E&M visit groups did not differ significantly, providers could consider TCM or E&M as post-discharge care alternatives to TCP based on patient needs.

In the research conducted by Kripalani et al. [32], risk prediction models were developed specifically for diseases like acute myocardial infarction, heart failure, stroke, and COPD, capable of classifying the patients into low- and high-risk groups. The project BOOST (Better Outcomes for Older Adults through Safe Transitions), led by the Society of Hospital Medicine [33], used a risk assessment tool that assessed eight patient factors contributing to readmission risk [34]. The present study, however, focused on several health conditions and had similar implementation approaches as other programs in the literature, such as having dedicated team members take care of patients, focusing post-discharge care attention on primary diagnoses related to hospitalizations, care coordination, medication reconciliation, and education with follow-up visits. These common structured elements may be important for TCP success.

### 4.4. Findings Concerning Cost

The finding that the TCP program reduces total care costs has a significant bearing in terms of hospital management of these specific disease subgroups of patients. Furthermore, the care costs were lower for the TCP-Graduate group compared to the DNG group regardless of conditions for hospitalization except for COPD, where episodes were nevertheless smaller in number than the other conditions. The savings were substantial not only in terms of actual costs but also in terms of the percentage of episodes with costs greater than target prices. With respect to financial penalties of up to 1% of Medicare reimbursements, at the scale of our study, the savings from the TCP program were unlikely to cover the costs of the TCP program. However, our healthcare system is continuing to encourage participation in and growth of the TCP program. We now take all comers at high risk of readmission per LACE score (which takes into account patient factors such as length of stay, acuity of the admission, comorbidities, and emergency department use in the duration of six months before admission) and provider discretion. Moreover, as we continue to grow our at-risk products and other high-risk contracts and launch our own in-house insurance product, we anticipate that the TCP program will become cost-effective.

### 4.5. Future Study for Increasing TCP Graduations

Despite these promising findings, the number of patients who successfully graduated from the TCP was much smaller (6.1%) than the DNG group or its subgroups. The underlying reasons for such a low graduation rate are largely unknown from the data available for the present study. Potential factors for low enrollment in or graduation from the TCP could be the inadequacy of the TCP to cater to the range of needs of the patients [35], patient characteristics such as age, dementia, frailty, and carer status, which may make the program less suitable for the patient [36], or issues with integrating the TCP with existing services [37]. Identification of these factors will help develop interventions designed to promote enrollment and graduation and evaluate the program in comparison with other programs in terms of program contents, post-discharge outcomes, and costs across several disease-related groups for index hospitalizations.

### 4.6. Strengths and Limitations

Our study has the following strengths. First, our study comprehensively evaluated and described the TCP in comparison with other programs in terms of program contents, post-discharge outcomes, and costs across several disease-related groups for index hospitalizations. Second, the data on outcomes and costs were rigorously extracted and validated from electronic health records, and Medicare claims data were provided by CMS through participation in the Bundled Payments for Care Improvement Advanced (BPCI-A) model. Third, our study findings are consistent with those reported in the literature, even though the data were collected from a regional tertiary referral hospital.

Study limitations are as follows. This was an observational study where the post-discharge care was not randomly assigned. Therefore, the results should be interpreted with consideration for the possibility of selection bias. Various patient characteristics like age, gender, race, marital status, admission type, and source of admission were not collected during the study, which can also affect the study results. As per Morkisch et al. [7], patients over 65 years old are more likely to undergo frequent readmissions classified as high-risk conditions. The sample size was relatively small and varied from group to group. Concerning the cost of care, the tests of significance to compare costs between the TCP-Graduated group and other groups were not able to be performed as individual-level cost data were not available. The analysis did not account for multiple comparisons. It is possible that some patients were not offered the TCP program for diverse reasons, including being discharged prior to being contacted, not being present in the hospital room when the call was made, and/or being unable to answer the phone or the phone not working. We do not have data on these specific reasons. Finally, the data provided is from a single institutional medical center at Prisma Health; therefore, the findings may not be generalizable to other medical centers.

## 5. Conclusions

In conclusion, the TCP had the potential for reducing hospital readmissions, total healthcare costs, and episodes exceeding target prices. These findings should be tested in further studies using more rigorous research designs and individual-level data. If found to be effective, TCPs will be instrumental in providing both high-quality and cost-effective care as more healthcare systems move to at-risk payment models. In the context of decreased Medicare reimbursements, TCPs may also allow viable and sustainable business models for healthcare systems that are increasingly operating at a deficit.

## Figures and Tables

**Table 1:** Number of Episodes by Post-Discharge Care Groups and Conditions for Hospitalization.

	Conditions for Hospitalization: n (%) *					
Group	CHF	COPD	PNE	Sepsis	PCI	All
Graduated	24 (11.2%)	11 (13.9%)	9 (5.2%)	22 (3.4%)	19 (6.9%)	85 (6.1%)
Did Not Graduate	191 (88.8%)	68 (86.1%)	165 (94.8%)	628 (96.6%)	258 (93.1%)	1310 (93.9%)
Joined but DNG	19 (8.8%)	0 (0.0%)	6 (3.4%)	21 (3.2%)	14 (5.1%)	60 (4.3%)
TCM Visit	8 (3.7%)	6 (7.6%)	7 (4.0%)	48 (7.4%)	29 (10.5%)	98 (7.0%)
E&M Visit	54 (25.1%)	26 (32.9%)	36 (20.7%)	118 (18.2%)	79 (28.5)	313 (22.4%)
No follow-up	110 (51.2%)	36 (45.6%)	116 (66.7%)	441 (67.8%)	136 (49.1%)	839 (60.1%)
Total	215	79	174	650	277	1395

Note: * Percentages are calculated compared to the total number of episodes. Abbreviations: DNG (Did Not Graduate), TCM (Transitional Care Management), E&M (Evaluation and Management), CHF (Chronic Heart Failure), COPD (Chronic Obstructive Pulmonary Disease), PNE (Pneumonia), PCI (Percutaneous Coronary Intervention).

**Table 2:** Comparisons of post-discharge utilization outcomes based on total episodes combining all conditions for hospitalization.

	Post-Discharge Utilization Outcome (Percentages, *p*-Value)			
Comparison	30-Day Readmission	90-Day Readmission	30-Day ED Visit	90-Day ED Visit
TCP-Graduated (N = 85) vs. DNG (N = 1310)	**7.1% vs. 14.9% (*p* = 0.046)**	**15.5% vs. 26.3% (*p* = 0.025)**	9.4% vs. 11.2% (*p* = 0.607)	20.0% vs. 21.9% (*p* = 0.680)
TCP-Graduated (N = 85) vs. Joined but DNG (N = 60)	**7.1% vs. 23.3% (*p* < 0.005)**	**15.5% vs. 41.7% (*p* < 0.001)**	9.4% vs. 18.3% (*p* = 0.117)	20.0% vs. 28.3% (*p* = 0.243)
TCP-Graduated (N = 85) vs. TCM visit (N = 98)	7.1% vs. 12.2% (*p* = 0.240)	15.5% vs. 20.4% (*p* = 0.369)	9.4% vs. 10.2% (*p* = 0.858)	20.0% vs. 20.4% (*p* = 0.945)
TCP-Graduated (N = 85) vs. E&M visit (N = 313)	7.1% vs. 12.5% (*p* = 0.163)	15.5% vs. 24.0% (*p* = 0.088)	9.4% vs. 10.2% (*p* = 0.858)	20.0% vs. 20.8% (*p* = 0.877)
TCP-Graduated (N = 85) vs. No follow-up (N = 839)	**7.1% vs. 15.5% (*p* = 0.036)**	**15.5% vs. 26.7% (*p* = 0.022)**	9.4% vs. 11.2% (*p* = 0.615)	20.0% vs. 22.1% (*p* = 0.663)
TCM visit (N = 98) vs. No follow-up (N = 839)	12.2% vs. 15.5% (*p* = 0.396)	20.4% vs. 26.7% (*p* = 0.179)	10.2% vs. 11.2% (*p* = 0.766)	20.4% vs. 22.1% (*p* = 0.710)
E&M visit (N = 313) vs. No follow-up (N = 839)	12.5% vs. 15.5% (*p* = 0.195)	24.0% vs. 26.7% (*p* = 0.346)	10.2% vs. 11.2% (*p* = 0.635)	20.8% vs.22.1% (*p* = 0.638)

Note: Significant results are indicated in boldface. Abbreviations: DNG (Did Not Graduate), TCP (Transition Care Program), TCM (Transitional Care Management), E&M (Evaluation and Management).

**Table 3:** Post-Discharge Utilization Outcome Comparisons Based on Follow-Up Care Types Stratified by Conditions for Hospitalization.

	Post-Discharge Utilization Outcome (Percentages, *p*-Value)			
Chronic Heart Failure (CHF)				
Comparison	30-Day Readmission	90-Day Readmission	30-Day ED Visit	90-Day ED Visit
TCP-Graduated (N = 24) vs. DNG (N = 191)	8.3% vs. 16.8% (*p* = 0.384)	20.8% vs. 31.4% (*p* = 0.287)	12.5% vs. 12% (*p* = 1.000)	25% vs. 23.6% (*p* = 0.876)
TCP-Graduated (N = 24) vs. Joined but DNG (N = 19)	**8.3% vs. 42.1% (*p* = 0.013)**	**20.8% vs. 57.9% (*p* = 0.013)**	12.5% vs. 21.1% (*p* = 0.680)	25% vs. 26.3% (*p* = 1.000)
TCP-Graduated (N = 24) vs. TCM visit (N = 8)	8.3% vs. 0% (*p* = 1.000)	20.8% vs. 12.5% (*p* = 1.000)	12.5% vs. 0% (*p* = 0.555)	25% vs. 12.5% (*p* = 0.646)
TCP-Graduated (N = 24) vs. E&M visit (N = 54)	8.3% vs. 13% (*p* = 0.713)	20.8% vs. 27.8% (*p* = 0.517)	12.5% vs. 14.8% (*p* = 1.000)	25% vs. 25.9% (*p* = 0.931)
TCP-Graduated (N = 24) vs. No follow-up (N = 110)	8.3% vs. 15.5% (*p* = 0.525)	20.8% vs. 30% (*p* = 0.367)	12.5% vs. 10% (*p* = 0.716)	25% vs. 22.7% (*p* = 0.811)
TCM visit (N = 8) vs. No follow-up (N = 110)	0% vs. 15.5% (*p* = 0.229)	12.5% vs. 30% (*p* = 0.436)	0% vs. 10% (*p* = 0.348)	12.5% vs. 22.7% (*p* = 0.683)
E&M visit (N = 54) vs. No follow-up (N = 110)	13% vs. 15.5% (*p* = 0.671)	27.8% vs. 30% (*p* = 0.769)	14.8% vs. 10% (*p* = 0.365)	25.9% vs. 22.7% (*p* = 0.651)
Chronic Obstructive Pulmonary Disease (COPD)				
Comparison	30-Day Readmission	90-Day Readmission	30-Day ED visit	90-Day ED visit
TCP-Graduated (N = 11) vs. DNG (N = 68)	9.1% vs. 14.7% (*p* = 1.000)	18.2% vs. 27.9% (*p* = 0.718)	0% vs. 10.3% (*p* = 0.265)	9.1% vs. 20.6% (*p* = 0.680)
TCP-Graduated (N = 11) vs. Joined but DNG (N = 0)	9.1% vs. 0% (-)	18.2% vs. 0% (-)	0% vs. 0% (-)	9.1% vs. 0% (-)
TCP-Graduated (N = 11) vs. TCM visit (N = 6)	9.1% vs. 0% (*p* = 1.000)	18.2% vs. 16.7% (*p* = 1.000)	0% vs. 0%	9.1% vs. 0% (*p* = 1.000)
TCP-Graduated (N = 11) vs. E&M visit (N = 26)	9.1% vs. 15.4% (*p* = 1.000)	18.2% vs. 23.1% (*p* = 1.000)	0% vs. 7.7% (*p* = 1.000)	9.1% vs. 19.2% (*p* = 0.646)
TCP-Graduated (N = 11) vs. No follow-up (N = 36)	9.1% vs. 16.7% (*p* = 1.000)	18.2% vs. 33.3% (*p* = 0.464)	0% vs. 13.9% (*p* = 0.322)	9.1% vs. 25% (*p* = 0.413)
TCM visit (N = 6) vs. No follow-up (N = 36)	0% vs. 16.7% (*p* = 0.280)	16.7% vs. 33.3% (*p* = 0.647)	0% vs. 13.9% (*p* = 1.000)	0% vs. 25% (*p* = 0.312)
E&M visit (N = 26) vs. No follow-up (N = 36)	15.4% vs. 16.7% (*p* = 1.000)	23.1% vs. 33.3% (*p* = 0.380)	7.7% vs. 13.9% (*p* = 0.689)	19.2% vs. 25% (*p* = 0.592)
Pneumonia (PNE)				
Comparison	30-Day Readmission	90-Day Readmission	30-Day ED visit	90-Day ED visit
TCP-Graduated (N = 9) vs. DNG (N = 165)	0% vs. 16.4% (*p* = 0.187)	0% vs. 26.7% (*p* = 0.073)	0% vs. 11.5% (*p* = 0.281)	0% vs. 21.2% (*p* = 0.122)
TCP-Graduated (N = 9) vs. Joined but DNG (N = 6)	0% vs. 0% (-)	0% vs. 33.3% (*p* = 0.143)	0% vs. 33.3% (*p* = 0.143)	0% vs. 33.3% (*p* = 0.143)
TCP-Graduated (N = 9) vs. TCM visit (N = 7)	0% vs. 28.6% (*p* = 0.175)	0% vs. 28.6% (*p* = 0.175)	0% vs. 0% (-)	0% vs. 0% (-)
TCP-Graduated (N = 9) vs. E&M visit (N = 36)	0% vs. 19.4% (*p* = 0.150)	0% vs. 25% (*p* = 0.094)	0% vs. 8.3% (*p* = 1.000)	0% vs. 19.4% (*p* = 0.150)
TCP-Graduated (N = 9) vs. No follow-up (N = 116)	0% vs. 15.5% (*p* = 0.201)	0% vs. 26.7% (*p* = 0.074)	0% vs. 12.1% (*p* = 0.269)	0% vs. 22.4% (*p* = 0.111)
TCM visit (N = 7) vs. No follow-up (N = 116)	28.6% vs. 15.5% (*p* = 0.318)	28.6% vs. 26.7% (*p* = 1.000)	0% vs. 12.1% (*p* = 0.329)	0% vs. 22.4% (*p* = 0.158)
E&M visit (N = 36) vs. No follow-up (N = 116)	19.4% vs. 15.5% (*p* = 0.579)	25% vs. 26.7% (*p* = 0.837)	8.3% vs. 12.1% (*p* = 0.763)	19.4% vs. 22.4% (*p* = 0.706)
Sepsis				
Comparison	30-Day Readmission	90-Day Readmission	30-Day ED visit	90-Day ED visit
TCP-Graduated (N = 22) vs. DNG (N = 628)	9.1% vs. 15.3% (*p* = 0.557)	18.2% vs. 27.7% (*p* = 0.325)	0% vs. 10% (*p* = 0.118)	13.6% vs. 20.4% (*p* = 0.593)
TCP-Graduated (N = 22) vs. Joined but DNG (N = 21)	9.1% vs. 14.3% (*p* = 0.664)	18.2% vs. 33.3% (*p* = 0.255)	0% vs. 14.3% (*p* = 0.108)	13.6% vs. 23.8% (*p* = 0.457)
TCP-Graduated (N = 22) vs. TCM visit (N = 48)	9.1% vs. 14.6% (*p* = 0.709)	18.2% vs. 27.1% (*p* = 0.420)	0% vs. 6.3% (*p* = 0.547)	13.6% vs. 12.5% (*p* = 1.000)
TCP-Graduated (N = 22) vs. E&M visit (N = 118)	9.1% vs. 12.7% (*p* = 1.000)	18.2% vs. 28.8% (*p* = 0.303)	0% vs. 7.6% (*p* = 0.181)	13.6% vs. 17.8% (*p* = 0.766)
TCP-Graduated (N = 22) vs. No follow-up (N = 441)	9.1% vs. 16.1% (*p* = 0.552)	18.2% vs. 27.2% (*p* = 0.351)	0% vs. 10.9% (*p* = 0.102)	13.6% vs. 21.8% (*p* = 0.593)
TCM visit (N = 48) vs. No follow-up (N = 441)	14.6% vs. 16.1% (*p* = 0.785)	27.1% vs. 27.2% (*p* = 0.985)	6.3% vs. 10.9% (*p* = 0.318)	12.5% vs. 21.8% (*p* = 0.133)
E&M visit (N = 118) vs. No follow-up (N = 441)	12.7% vs. 16.1% (*p* = 0.365)	28.8% vs. 27.2% (*p* = 0.729)	7.6% vs. 10.9% (*p* = 0.299)	17.8% vs. 21.8% (*p* = 0.346)
Percutaneous Coronary Intervention (PCI)				
Comparison	30-Day Readmission	90-Day Readmission	30-Day ED visit	90-Day ED visit
TCP-Graduated (N = 19) vs. DNG (N = 258)	5.3% vs. 11.6% (*p* = 0.706)	10.5% vs. 18.2% (*p* = 0.542)	26.3% vs. 13.6% (*p* = 0.167)	36.8% vs. 25.2% (*p* = 0.282)
TCP-Graduated (N = 19) vs. Joined but DNG (N = 14)	5.3% vs. 21.4% (*p* = 0.288)	10.5% vs. 35.7% (*p* = 0.106)	26.3% vs. 14.3% (*p* = 0.670)	36.8% vs. 35.7% (*p* = 0.947)
TCP-Graduated (N = 19) vs. TCM visit (N = 29)	5.3% vs. 10.3% (*p* = 1.000)	10.5% vs. 10.3% (*p* = 1.000)	26.3% vs. 24.1% (*p* = 1.000)	36.8% vs. 44.8% (*p* = 0.583)
TCP-Graduated (N = 19) vs. E&M visit (N = 79)	5.3% vs. 7.6% (*p* = 1.000)	10.5% vs. 13.9% (*p* = 1.000)	26.3% vs. 12.7% (*p* = 0.160)	36.8% vs. 22.8% (*p* = 0.245)
TCP-Graduated (N = 19) vs. No follow-up (N = 136)	5.3% vs. 13.2% (*p* = 0.471)	10.5% vs. 20.6% (*p* = 0.372)	26.3% vs. 11.8% (*p* = 0.142)	36.8% vs. 21.3% (*p* = 0.151)
TCM visit (N = 29) vs. No follow-up (N = 136)	10.3% vs. 13.2% (*p* = 1.000)	10.3% vs. 20.6% (*p* = 0.200)	24.1% vs. 11.8% (*p* = 0.134)	**44.8% vs. 21.3% (*p* < 0.01)**
E&M visit (N = 79) vs. No follow-up (N = 136)	7.6% vs. 13.2% (*p* = 0.205)	13.9% vs. 20.6% (*p* = 0.222)	12.7% vs. 11.8% (*p* = 0.846)	22.8% vs. 21.3% (*p* = 0.803)

Note: Significant results are indicated in boldface. Abbreviations: TCP (Transition Care Program), TCM (Transitional Care Management), E&M (Evaluation and Management), PCP (Primary Care Provider).

**Table 4:** Average Total Cost per Episode Stratified by Conditions for Hospitalizations and Post-Discharge Care Groups.

Condition	Care Groups	N	Total Cost	1–30 Day Cost	31–90 Day Cost	%Episodes over Target Price	*p*-Value *
CHF	TCP-Graduated	24	USD 26,017	USD 6054	USD 11,654	41.7%	
	Did Not Graduate	191	USD 21,531	USD 7799	USD 5848	39.8%	0.860
	Joined but DNG	19	USD 20,825	USD 9973	USD 3794	47.4%	0.708
	TCM Visit With PCP	8	USD 20,279	USD 8769	USD 3740	37.5%	1.000
	E&M Visit With PCP	54	USD 21,866	USD 5905	USD 8235	37.0%	0.698
	No Follow-Up	110	USD 21,579	USD 8282	USD 5184	40.9%	0.946
	Total	215	USD 22,031	USD 7604	USD 6496	40.0%	
COPD	TCP-Graduated	11	USD 12,565	USD 3766	USD 2597	27.3%	
	Did Not Graduate	68	USD 15,374	USD 5220	USD 4219	41.2%	0.513
	Joined but DNG	0	-	-	-	-	-
	TCM Visit With PCP	6	USD 13,102	USD 2658	USD 4733	33.3%	1.000
	E&M Visit With PCP	26	USD 10,669	USD 2031	USD 2758	19.2%	0.672
	No Follow-Up	36	USD 19,151	USD 7951	USD 5189	58.3%	0.071
	Total	79	USD 14,983	USD 5018	USD 3993	39.2%	
PNE	TCP-Graduated	9	USD 12,988	USD 2432	USD 3318	22.2%	
	Did Not Graduate	165	USD 21,369	USD 8111	USD 5373	43.0%	0.307
	Joined but DNG	6	USD 20,740	USD 2237	USD 11,179	50.0%	0.329
	TCM Visit With PCP	7	USD 21,450	USD 8000	USD 6544	28.6%	1.000
	E&M Visit With PCP	36	USD 20,769	USD 5967	USD 7387	36.1%	0.695
	No Follow-Up	116	USD 21,583	USD 9087	USD 4376	45.7%	0.296
	Total	174	USD 20,936	USD 7817	USD 5266	42.0%	
Sepsis	TCP-Graduated	22	USD 24,754	USD 6231	USD 5809	13.6%	
	Did Not Graduate	628	USD 32,973	USD 12,028	USD 7644	36.0%	**0.031**
	Joined but DNG	21	USD 23,992	USD 5801	USD 6971	19.0%	0.698
	TCM Visit With PCP	48	USD 23,299	USD 5383	USD 6835	18.8%	0.741
	E&M Visit With PCP	118	USD 27,501	USD 6887	USD 8178	27.1%	0.180
	No Follow-Up	441	USD 35,917	USD 14,423	USD 7622	41.0%	**0.010**
	Total	650	USD 32,695	USD 11,831	USD 7582	36.0%	
PCI	TCP-Graduated	19	USD 25,430	USD 2964	USD 3071	21.1%	
	Did Not Graduate	258	USD 31,466	USD 5424	USD 5694	29.8%	0.416
	Joined but DNG	14	USD 38,051	USD 10,143	USD 5848	50.0%	0.136
	TCM Visit With PCP	29	USD 28,486	USD 4405	USD 4756	24.1%	1.000
	E&M Visit With PCP	79	USD 27,481	USD 2578	USD 5361	19.0%	1.000
	No Follow-Up	136	USD 33,739	USD 6809	USD 6072	36.0%	0.197
	Total	277	USD 31052	USD 5255	USD 5514	30.0%	
All	TCP-Graduated	85	USD 22,439	USD 4730	USD 6168	25.9%	
	Did Not Graduate	1310	USD 28,633	USD 9264	USD 6534	36.6%	**0.018**
	Joined but DNG	60	USD 25,944	USD 7779	USD 6124	38.3%	0.110
	TCM Visit With PCP	98	USD 23,831	USD 5390	USD 5818	23.5%	0.705
	E&M Visit With PCP	313	USD 24,351	USD 5121	USD 6936	27.2%	0.814
	No Follow-Up	839	USD 30,983	USD 11,368	USD 6498	41.6%	**0.005**
	Total	1395	USD 28,255	USD 8987	USD 6512	36.3%	

Note: * Comparison of % Episodes over Target Price with the TCP-Graduated group. Significant results are indicated in boldface. Abbreviations: DNG (Did Not Graduate), TCP (Transition Care Program), TCM (Transitional Care Management), E&M (Evaluation and Management), CHF (Chronic Heart Failure), COPD (Chronic Obstructive Pulmonary Disease), PNE (Pneumonia), PCI (Percutaneous Coronary Intervention).

## Data Availability

The data presented in this study are available on reasonable request from the corresponding author.

## Abbreviations

AMI: Acute Myocardial Infarction; BOOST: Better Outcomes for Older adults through Safe Transitions; BPCI: Bundled Payments for Care Improvement; CABG: Coronary Artery Bypass Graft; CDP: Comprehensive Discharge Planning; CHF: Chronic Heart Failure; CMS: Center for Medicare & Medicaid Services; COPD: Chronic Obstructive Pulmonary Disease; CTI: Care Transitions Initiative; DNG: Did Not Graduate; ED: Emergency Department; EHR: Electronic Health Record; E&M: Evaluation and Management; HF: Heart Failure; HK: Hip/knee arthroplasty; PCI: Percutaneous Coronary Intervention; PCP: Primary Care Physician; PNE: Pneumonia; RED: Reengineering Discharge; SDoH: Social Determinants of Health; TCM: Transitional Care Management; TCP: Transitional Care Program.

## References

[1] Dharmarajan K., Hsieh A.F., Lin Z., Bueno H., Ross J.S., Horwitz L.I., Barreto-Filho J.A., Kim N., Bernheim S.M., Suter L.G. (2013). Diagnoses and timing of 30-day readmissions after hospitalization for heart failure, acute myocardial infarction, or pneumonia. JAMA.

[2] Lahijanian B., Alvarado M. (2021). Care Strategies for Reducing Hospital Readmissions Using Stochastic Programming. Healthcare.

[3] Jencks S.F., Williams M.V., Coleman E.A. (2009). Rehospitalizations among patients in the Medicare fee-for-service program. N. Eng. J. Med..

[4] Budnitz D.S., Lovegrove M.C., Shehab N., Richards C.L. (2011). Emergency hospitalizations for adverse drug events in older Americans. N. Eng. J. Med..

[5] Rennke S., Ranji S.R. (2015). Transitional care strategies from hospital to home: A review for the neurohospitalist. Neurohospitalist.

[6] Low L.L., Vasanwala F.F., Ng L.B., Chen C., Lee K.H., Tan S.Y. (2015). Effectiveness of a transitional home care program in reducing acute hospital utilization: A quasi-experimental study. BMC Health Serv. Res..

[7] Morkisch N., Upegui-Arango L.D., Cardona M.I., van den Heuvel D., Rimmele M., Sieber C.C., Freiberger E. (2020). Components of the transitional care model (TCM) to reduce readmission in geriatric patients: A systematic review. BMC Geriatr..

[8] Hayes S.L., Salzberg C.A., McCarthy D., Radley D.C., Abrams M.K., Shah T., Anderson G.F. (2016). High-Need, High-Cost Patients: Who Are They and How Do They Use Health Care? A Population-Based Comparison of Demographics, Health Care Use, and Expenditures. Issue Brief (Commonw. Fund).

[9] Hong C.S., Siegel A.L., Ferris T.G. (2014). Caring for high-need, high-cost patients: What makes for a successful care management program?. Issue Brief (Commonw. Fund).

[10] Long P.M., Abrams A., Milstein G., Anderson K., Apton L., Dahlberg M.L., Whicher D. (2017). Effective Care for High-Need Patients: Opportunities for Improving Outcomes, Value, and Health.

[11] Bailey J.E., Surbhi S., Wan J.Y., Munshi K.D., Waters T.M., Binkley B.L., Ugwueke M.O., Graetz I. (2019). Effect of Intensive Interdisciplinary Transitional Care for High-Need, High-Cost Patients on Quality, Outcomes, and Costs: A Quasi-Experimental Study. J. Gen. Intern. Med..

[12] Ba H.M., Son Y.J., Lee K., Kim B.H. (2020). Transitional Care Interventions for Patients with Heart Failure: An Integrative Review. Int. J. Environ. Res. Public Health.

[13] Koehler B.E., Richter K.M., Youngblood L., Cohen B.A., Prengler I.D., Cheng D., Masica A.L. (2009). Reduction of 30-Day Postdischarge Hospital Readmission or Emergency Department (ED) Visit Rates in High-Risk Elderly Medical Patients through Delivery of a Targeted Care Bundle. J. Hosp. Med..

[14] Le Berre M., Maimon G., Sourial N., Gueriton M., Vedel I. (2017). Impact of Transitional Care Services for Chronically Ill Older Patients: A Systematic Evidence Review. J. Am. Geriatr. Soc..

[15] Pfuntner A., Wier L.M., Stocks C. (2010). Most Frequent Conditions in U.S. Hospitals. http://www.hcup-us.ahrq.gov/reports/statbriefs/sb148.pdf.

[16] Pfuntner A., Wier L.M., Elixhauser A. (2006). Overview of Hospital Stays in the United States, 2010. Healthcare Cost and Utilization Project (HCUP) Statistical Briefs.

[17] Ford E.S., Croft J.B., Mannino D.M., Wheaton A.G., Zhang X., Giles W.H. (2013). COPD surveillance—United States, 1999–2011. Chest.

[18] Press V.G., Arora V.M., Shah L.M., Lewis S.L., Charbeneau J., Naureckas E.T., Krishnan J.A. (2012). Teaching the use of respiratory inhalers to hospitalized patients with asthma or COPD: A randomized trial. J. Gen. Intern. Med..

[19] Mohr N.M., Zebrowski A.M., Gaieski D.F., Buckler D.G., Carr B.G. (2020). Inpatient hospital performance is associated with post-discharge sepsis mortality. Crit. Care.

[20] Shankar-Hari M., Harrison D.A., Ferrando-Vivas P., Rubenfeld G.D., Rowan K. (2019). Risk Factors at Index Hospitalization Associated With Longer-term Mortality in Adult Sepsis Survivors. JAMA Netw. Open.

[21] Rashedi S., Tavolinejad H., Kazemian S., Mardani M., Masoudi M., Masoudkabir F., Haghjoo M. (2022). Efficacy and safety of same-day discharge after atrial fibrillation ablation: A systematic review and meta-analysis. Clin. Cardiol..

[22] Vedel I., Khanassov V. (2015). Transitional Care for Patients with Congestive Heart Failure: A Systematic Review and Meta-Analysis. Ann. Fam. Med..

[23] Jo H.S., Jeong S., Kim W.J., Park S., Yu S.A. (2020). Development of a Transitional Care Model Program for Patients with Pneumonia, Asthma, and Chronic Obstructive Pulmonary Disease: In-depth Interviews with Readmitted Patients. J. Korean Med. Sci..

[24] Fonarow G.C., Smith E.E., Reeves M.J., Pan W., Olson D., Hernandez A.F., Peterson E.D., Schwamm L.H. (2011). Hospital-level variation in mortality and rehospitalization for medicare beneficiaries with acute ischemic stroke. Stroke.

[25] Ota K.S., Beutler D.S., Gerkin R.D., Weiss J.L., Loli A.I. (2013). Physician-directed heart failure transitional care program: A retrospective case review. J. Clin. Med. Res..

[26] Ford E.S. (2015). Hospital Discharges, Readmissions, and ED Visits for COPD or Bronchiectasis Among US Adults Findings from the Nationwide Inpatient Sample 2001–2012 and Nationwide Emergency Department Sample 2006–2011. Chest.

[27] Venkatesh A.K., Dai Y., Ross J.S., Schuur J.D., Capp R., Krumholz H.M. (2015). Variation in US Hospital Emergency Department Admission Rates by Clinical Condition. Med. Care.

[28] Coleman E., Parry C., Chalmers S., Min S. (2006). The care transitions intervention—Results of a randomized controlled trial. Arch. Intern. Med..

[29] Jack B., Chetty V., Anthony D., Greenwald J., Sanchez G., Johnson A., Forsythe S., O’Donnell J., Paasche-Orlow M., Manasseh C. (2009). A Reengineered Hospital Discharge Program to Decrease Rehospitalization A Randomized Trial. Ann. Intern. Med..

[30] Naylor M., Brooten D., Jones R., Lavizzomourey R., Mezey M., Pauly M. (1994). Comprehensive Discharge Planning for the Hospitalized Elderly—A Randomized Clinical-Trial. Ann. Intern. Med..

[31] Kripalani S., Chen G., Ciampa P., Theobald C., Cao A., McBride M., Dittus R., Speroff T. (2019). A transition care coordinator model reduces hospital readmissions and costs. Contemp. Clin. Trials.

[32] Kripalani S., Theobald C.N., Anctil B., Vasilevskis E.E. (2014). Reducing hospital readmission rates: Current strategies and future directions. Annu. Rev. Med..

[33] Coffey C., Greenwald J., Budnitz T., Williams M. (2013). Project BOOST^®^ Implementation Guide (Second Edition).

[34] Hansen L.O., Greenwald J.L., Budnitz T., Howell E., Halasyamani L., Maynard G., Vidyarthi A., Coleman E.A., Williams M.V. (2013). Project BOOST: Effectiveness of a multihospital effort to reduce rehospitalization. J. Hosp. Med..

[35] Giles L.C., Halbert J.A., Gray L.C., Cameron I.D., Crotty M. (2009). The distribution of health services for older people in Australia: Where does transition care fit?. Aust. Health Rev..

[36] Cations M., Lang C., Crotty M., Wesselingh S., Whitehead C., Inacio M.C. (2020). Factors associated with success in transition care services among older people in Australia. BMC Geriatr..

[37] Masters S., Halbert J., Crotty M., Cheney F. (2008). Innovations in Aged Care: What are the first quality reports from the Transition Care Program in Australia telling us?. Australas. J. Ageing.

